# An Enhanced Electromagnetic Manipulation System with a Large Workspace, High-Gradient Magnetic Actuation, and Efficient Thermal Management

**DOI:** 10.3390/mi17070810

**Published:** 2026-07-02

**Authors:** Junkai Zhang, Zerui Li, Yukun Zhong, Aaiza Gul, U Kei Cheang

**Affiliations:** 1 Department of Mechanical and Energy Engineering, Southern University of Science and Technology, Shenzhen 518055, China; 12231131@mail.sustech.edu.cn (J.Z.); 12431172@mail.sustech.edu.cn (Z.L.); 12131098@mail.sustech.edu.cn (Y.Z.); 2 State Key Laboratory of Fluid Power & Mechatronic Systems, School of Mechanical Engineering, Zhejiang University, Hangzhou 310027, China; aaizagul@gmail.com; 3 Shenzhen Key Laboratory of Biomimetic Robotics and Intelligent Systems, Southern University of Science and Technology, Shenzhen 518055, China; 4 Guangdong Provincial Key Laboratory of Human-Augmentation and Rehabilitation Robotics in Universities, Southern University of Science and Technology, Shenzhen 518055, China

**Keywords:** magnetic manipulation, enhanced electromagnet, magnetic field optimization, FEM simulation, micro/nanorobotics

## Abstract

Magnetic actuation is a fundamental enabling technology for micro/nanorobotics and biomedical manipulation. However, the trade-off between magnetic field gradient, usable workspace, and efficient heat dissipation often conflicts and constrains its performance. Here, we present an enhanced electromagnetic manipulation system (EEMS) based on a compact, high-efficiency magnetic circuit and an optimized six-electromagnet configuration. By integrating high-permeability structural components and employing finite-element-based optimization, the system achieves a spherical workspace of 106 mm in diameter while maintaining strong and spatially controllable magnetic fields. Experimental results demonstrate magnetic flux densities up to 300 mT and a magnetic field gradient up to 9.5 T/m within the workspace, with a central magnetic field gradient of approximately 2 T/m under continuous operation at 3 A. Thermal simulations and measurements confirm safe operation below human body temperature without active cooling. Magnetic manipulation experiments in viscous environments further validate precise motion control and force balancing, highlighting the system’s potential for advanced magnetic manipulation and intelligent microrobotic applications.

## 1. Introduction

With the rapid advancement of cutting-edge fields such as interventional medicine, targeted therapy, and micro/nano manipulation, there is an increasingly urgent demand for technologies capable of precise, remote actuation within complex biological environments [1,2]. Magnetic field actuation, with its rapid response, high-precision control, excellent biocompatibility, and remote manipulation capabilities, is recognized as one of the most promising drive solutions in these domains [3]. In clinical practice, magnetic capsule endoscopes are already in use [4]. Magnetically driven guidewire and catheter systems, guided by external magnetic fields, have demonstrated significant potential in enhancing maneuvering precision and reducing iatrogenic injury during procedures like cardiovascular interventions [5]. Meanwhile, magnetically driven micro/nanorobots are being extensively explored for applications such as targeted drug delivery, cell manipulation, and minimally invasive surgery [6,7], with integrated motion control, imaging, and drug-loading capabilities emerging as current research hotspots [8]. Compared to single robots, swarms offer the advantages of collaborative capabilities and high payload capacity, and there have been many breakthroughs in research in this area in recent years [9,10,11,12,13]. The common physical foundation for these applications lies in the ability of external drive devices to generate magnetic fields with specific spatio-temporal distributions, including uniform fields, gradients, and rotations, within the target workspace, thereby applying controllable forces and torques to magnetized objects [14]. Core performance metrics in this field are directly linked to magnetic field characteristics: magnetic flux density determines the upper limit of applicable torque, influencing drive speed and load capacity; while magnetic field gradient strength determines the magnitude of force that can be applied, which is crucial for traction, capture, and precise positioning [15]. The dimensions of the workspace and magnetic field uniformity determine the system’s applicability and manipulation accuracy. Although permanent magnet arrays excel at generating high-intensity static fields, their magnetic field variation depends on the motion actuator, which lacks flexibility [16]. While traditional electromagnetic configurations like Helmholtz coils and Maxwell coils can achieve dynamic fields through current programming, their magnetic flux density and magnetic field gradient are often constrained by limited ampere-turns and low magnetic circuit efficiency. This makes it difficult to simultaneously meet the requirements of high intensity and high uniformity in larger spaces, presenting a key bottleneck in the development of high-performance magnetic drive systems [17]. To enhance key metrics of drive magnetic fields, researchers have explored multiple dimensions. At the macroscale, the multi-coil electromagnetic system developed by Li et al. for catheter navigation achieves complex magnetic field vector control in large spaces by optimizing coil arrangement and current combinations [18]. In the field of micro/nanorobotic swarm, work by Yang et al. demonstrated the use of rotating magnetic fields to drive micro-swimmers and the gradient of fields for collective control, respectively [19]. Overall, these systems represent significant progress, though the magnetic flux density achievable in such setups is subject to constraints related to coil heat dissipation and power density limits.

The magnetic manipulation system is the key equipment to control the motion, position, and functionality of micro/nanorobots. A core challenge remains balancing large working spaces with the generation of sufficiently strong and rapidly varying magnetic fields. Most existing research focuses on coil arrangement optimization and advanced control algorithms [20,21,22]. Some novel works combine the manipulator with the electromagnet to realize the magnetic field in a large workspace [23,24,25]. Some studies are very enlightening and combine the coils with the motor to simplify the system volume [26,27]. However, as Nelson et al. noted in their seminal review of magnetic systems [28], the key to the clinical application of this kind of system is to reduce the volume and power consumption of the equipment while ensuring the space and magnetic field flux and gradient. An effective approach to overcoming this limitation has emerged by focusing on magnetic circuit design itself, specifically, introducing high-permeability materials to guide and concentrate magnetic flux [29]. The study of Li et al. demonstrated that they used mathematical analysis and the finite element method to evaluate and quantify the magnetic field characteristics between the air gap and the cylindrical magnetic coupler [30]. Nevertheless, research remains insufficient regarding the design of compact, efficient composite magnetic circuit systems capable of simultaneously supporting both rotational and gradient drive modes for multi-pole, three-dimensional dynamic magnetic field generation. Specifically, there is a lack of research on how to synergistically maximize both magnetic flux density and magnetic field gradient intensity through refined magnetic circuit structure optimization while ensuring excellent working space and Joule heating efficiency remains a key scientific question awaiting in-depth exploration.

To address the limitations of existing magnetic field manipulation systems in balancing workspace dimensions and magnetic flux density, this study proposes an enhanced electromagnetic field configuration system. The system employs a novel electromagnet configuration based on FEM structural optimization, utilizing six electromagnets distributed along a rotating Cartesian coordinate system. The working space of this enhanced electromagnetic manipulation system (EEMS) is a sphere with a diameter of 106 mm. When using a pair of electromagnets, the magnetic flux density at the center of the working space can reach 70 mT, with a magnetic field gradient of 2 T/m. Within the working space, the maximum magnetic flux density can reach 300 mT, with a magnetic field gradient of 9.5 T/m. The power of a single coil at a 3 A maximum current is 126 W. Compared to previous studies, the Octomag by Kummer et al. has achieved a 130 mm workspace, 15 mT magnetic flux density in the center, and a 200 mT/m magnetic flux density gradient. The power of a single coil at a 20 A maximum current is 520 W, and its temperature rises to 60 °C during operation [29]. The system by Niu et al. achieved a 120 mm workspace, 40 mT magnetic flux density at the center, and a 250 mT/m magnetic flux density gradient. The power of a single coil at a 10 A maximum current is 320 W, and its temperature rises to 50 °C during operation [31]. The system by Li et al. achieved a 16 mm workspace, 124 mT magnetic flux density at the center, and a 20 T/m magnetic flux density gradient with 2D magnetic control. The power of a single coil at a 10 A maximum current is 500 W [32]. The EEMS demonstrates significant advancement in balancing workspace size and magnetic flux density. To determine the safe operating range for current parameters, we also evaluated the EEMS’s heat dissipation capability. The evaluation was based on an FEM-based thermal simulation model. According to the simulated temperature projections, we established a peak current of 3 A, under which the electromagnet must operate continuously for 10 min with a maximum temperature below normal human body temperature. Actual measurements yielded temperatures lower than the simulated projections, revealing the structural thermal conduction’s advantage in enhancing the electromagnet’s heat dissipation capability. Building upon the EEMS, we designed an experiment involving the motion of micro-magnetic objects within a viscous liquid environment. This experiment validated the EEMS’s precision and stability in generating and controlling spatial magnetic fields from multiple perspectives.

## 2. Materials and Methods

Simulations: Simulations were performed using COMSOL Multiphysics 6.2. Studies of the electromagnets’ magnetic fields simulation in Section 3.1.2 and Section 3.1.3 used the Magnetic Field (mf) interface. Studies of the electromagnet’s thermal simulation in Section 3.1.4 used the Heat Transfer (ht) interface, the Laminar Flow (spf) interface, and the Nonisothermal Flow (nitf) interface. Simulation parameters are shown in Supporting Information Table S1. In the electromagnet simulation, the air area was defined as a sphere with a diameter of 1000 mm, using a free tetrahedral mesh with a maximum element size of 35 mm and a minimum element size of 1.5 mm. The boundary conditions for the sphere are set as Infinite Element Implementation to simulate the physical behavior of the magnetic field naturally decaying to zero at infinity. A swept mesh with 5 layers is used for the Infinite Element Implementation. The electromagnet uses the Coil interface in the Static Magnetic Field module to simulate a multi-turn coil with a wire diameter of 1 mm and 2380 turns, uniformly distributed across the cross-section. The core electromagnet uses a free tetrahedral mesh with a maximum element size of 2.4 mm and a minimum element size of 1 mm. The working space is set to a free tetrahedral mesh with a maximum element size of 3 mm and a minimum element size of 1 mm. In the thermal simulation, the air domain is defined as a cylinder with a diameter and length of 400 mm. In the Heat Transfer interface, the boundary condition for the air domain is set to Open Boundary, with an initial temperature of 293.15 K. The surface of the electromagnet is set to a surface-to-ambient radiation boundary condition. In the Laminar Flow interface, the boundary condition for the air domain is set to Open Boundary, with an initial velocity field of 0 m/s, and gravity is included. The multiphysics coupling boundary condition is set to Nonisothermal Flow and applied to the air domain.

Hardware: Translation Stage, Thorlabs, Newton, NJ, the U.S., PLSXY 2D Motorized Translation Stage for Rigid Stands. Joystick: Thorlabs, MCMK3 3-Knob USB HID Joystick. Core board: ALIENTEK, Guangzhou, China, ATK-DMF407 STM32F407 core board. Drive board: ALIENTEK, ATK-PD6010D DC brush drive board. DC power supply: Huatai, Yangzhou, China, HAP09-250D Programmable DC power supply. Camera: MSHOT, Guangzhou, China, MD60 digital camera.

Motion control experiments: The flow channel was printed using a Bambu Lab, Shenzhen, China, A1 printer with a 0.2 mm nozzle and PLA Basic material. The chemicals used in motion experiments are: sodium alginate (SA; CAS 9005-38-3; Aladdin, Shanghai, China), polyvinyl pyrrolidone (PVP; (C_6_H_9_NO)_n_; CAS 9003-39-8; Aladdin, China), and Fe_3_O_4_ magnetic nanoparticles (Fe_3_O_4_; CAS 1317-61-9; 100 nm; Macklin, Shanghai, China).

## 3. Results

### 3.1. System Design

#### 3.1.1. Force and Torque Under a Magnetic Field

For objects without current flowing through them, magnetic properties can be introduced by embedding magnetic nanoparticles (MNPs) or by using magnetic materials. Magnetic objects experience magnetic force or torque in a magnetic field, which is described by the magnetic force formula as follows:

(1) $$F=\left(m\cdot \nabla \right)B$$

where m denotes the magnetic moment vector and B denotes the magnetic flux density vector. Nabla operator ∇ is the gradient operator.

The magnetic torque formula is given by the following:

(2) T=m×B

For the magnetic field within the working region, it can be considered a quasi-static field that obeys the Maxwell equations for static magnetic fields:

(3) ∇·B=0

(4) ∇×B=0

Substituting (3) and (4) into (1) yields the following:

(5) $$F=\left[\begin{array}{ccc} \frac{\partial B_{x}}{\partial x} & \frac{\partial B_{x}}{\partial y} & \frac{\partial B_{x}}{\partial z}\\ \frac{\partial B_{x}}{\partial y} & \frac{\partial B_{y}}{\partial y} & \frac{\partial B_{y}}{\partial z}\\ \frac{\partial B_{x}}{\partial z} & \frac{\partial B_{y}}{\partial z} & -(\frac{\partial B_{x}}{\partial x}+\frac{\partial B_{y}}{\partial y}) \end{array}\right]m$$

For the magnetic field generated by an electromagnet driven by six independently controlled currents, the relationship between magnetic flux density and current at a point P defined in the working space can be expressed in a matrix as

(6) $$B\left(P\right)=\sum_{i=1}^{6}{\tilde{B}}_{i}\left(P\right)I_{i}=\tilde{B}\left(P\right)I$$

where $P={\left[\begin{array}{ccc} x & y & z \end{array}\right]}^{T}$ denotes the position in the workspace,

$I={\left[\begin{array}{cccccc} I_{1} & I_{2} & I_{3} & I_{4} & I_{5} & I_{6} \end{array}\right]}^{T}$ is the current vector and

(7) $$\tilde{B}\left(P\right)=\left[\begin{array}{cccccc} {\tilde{B}}_{1}\left(P\right) & {\tilde{B}}_{2}\left(P\right) & , & \ldots & , & {\tilde{B}}_{6}\left(P\right) \end{array}\right]\in R^{3\times 6}$$

is the magnetic field generation matrix at position P.

Similarly, the magnetic field gradient is formulated as

(8) $$\nabla B\left(P\right)=\nabla \tilde{B}\left(P\right)I$$

where $\nabla \tilde{B}\left(P\right)\in R^{5\times 6}$ is the gradient generation matrix.

By combining the magnetic field and gradient terms, the complete actuation model becomes

(9) $$Y=\left[\begin{array}{c} B_{x}\\ B_{y}\\ B_{z}\\ \frac{\partial B_{x}}{\partial x}\\ \frac{\partial B_{x}}{\partial y}\\ \frac{\partial B_{x}}{\partial z}\\ \frac{\partial B_{y}}{\partial y}\\ \frac{\partial B_{y}}{\partial z} \end{array}\right]=\left[\begin{array}{ccc} {{\tilde{B}}_{1}\left(P\right)}_{x} & \ldots & {{\tilde{B}}_{6}\left(P\right)}_{x}\\ {{\tilde{B}}_{1}\left(P\right)}_{y} & \ldots & {{\tilde{B}}_{6}\left(P\right)}_{y}\\ {{\tilde{B}}_{1}\left(P\right)}_{z} & \ldots & {{\tilde{B}}_{6}\left(P\right)}_{z}\\ \frac{{{\tilde{B}}_{1}\left(P\right)}_{x}}{\partial x} & \ldots & \frac{{{\tilde{B}}_{6}\left(P\right)}_{x}}{\partial x}\\ \frac{{{\tilde{B}}_{1}\left(P\right)}_{x}}{\partial y} & \ldots & \frac{{{\tilde{B}}_{6}\left(P\right)}_{x}}{\partial y}\\ \frac{{{\tilde{B}}_{1}\left(P\right)}_{x}}{\partial z} & \ldots & \frac{{{\tilde{B}}_{6}\left(P\right)}_{x}}{\partial z}\\ \frac{{{\tilde{B}}_{1}\left(P\right)}_{y}}{\partial y} & \ldots & \frac{{{\tilde{B}}_{6}\left(P\right)}_{y}}{\partial y}\\ \frac{{{\tilde{B}}_{1}\left(P\right)}_{y}}{\partial z} & \ldots & \frac{{{\tilde{B}}_{6}\left(P\right)}_{y}}{\partial z} \end{array}\right]\left[\begin{array}{c} I_{1}\\ I_{2}\\ I_{3}\\ I_{4}\\ I_{5}\\ I_{6} \end{array}\right]=GI$$

where G ∈ $R^{8\times 6}$ is a magnetic actuation matrix.

The magnetic actuation vector contains eight independent magnetic quantities, including three magnetic field components and five independent gradient components. Since the EEMS consists of six independently driven electromagnets, the actuation matrix has dimensions of 8 × 6.

Because G is a non-square matrix, a direct inverse does not exist. Furthermore, depending on the target position in the workspace, the actuation matrix may become poorly conditioned or locally rank-deficient due to coupling between electromagnets. The actuation system is generally overdetermined and may become rank-deficient at certain locations; the driving current is obtained using the Moore–Penrose pseudoinverse:

(10) $$I=G^{†}Y$$

where

(11) $$G^{†}={\left(G^{T}G\right)}^{-1}G^{T}$$

for the full-column-rank case.

The pseudoinverse provides the minimum Euclidean norm solution $\min{\left\|I\right\|}_{2}$ subject to (9), thereby reducing unnecessary current consumption and Joule heating while maintaining the desired magnetic actuation performance.

#### 3.1.2. Design of the Electromagnet Structure

The six electromagnets of the enhanced electromagnetic manipulation system (EEMS) share the same configuration, distributed along the six directions of the positive and negative semi-axes of the Cartesian coordinate system. The top of each electromagnet’s core is positioned 53 mm from the working space origin, defining a working space as a spherical region with a diameter of 106 mm. To ensure sufficient space for mounting optical components above and the displacement platform’s electric drive below, the six electromagnets are rotated around the working space origin: first 45° clockwise along the z-axis, then 54.74° clockwise along the x-axis. After rotation, a cylindrical device mounting space with a diameter of approximately 50 mm is obtained along the z-axis, preventing physical interference or collision between the optical components, the displacement platform, and electromagnets. Among the three tip configurations in Figure S4, the cylindrical tip offers a better distal magnetic field gradient, the 60° chamfered tip offers a better proximal magnetic field gradient, and the conical tip falls between the two. However, considering practical application—where a camera must be mounted above the working area and a translation stage below it, the 60° chamfered tip minimizes physical interference between the electromagnet and other equipment. After comprehensive consideration, we selected the 60° chamfered tip. More detailed parameters are shown in Table 1.

**Figure 1:**
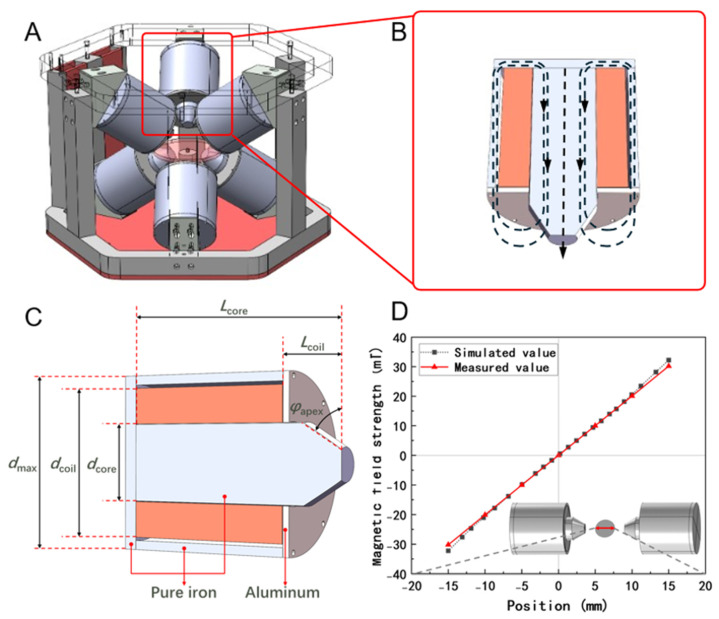
Enhanced electromagnetic manipulation system. (**A**) Schematic diagram of the EEMS. (**B**) Cross − sectional structure and magnetic flux line loop diagram of a single electromagnet. The dotted lines represent magnetic field lines, and the arrows indicate the direction of the magnetic field. (**C**) Key parameters and materials of a single electromagnet. (**D**) Simulated and actual measured magnetic flux density distribution near the origin along the central axis (axis of rotational symmetry) for a pair of electromagnets.

A partial perspective 3D structural diagram of EEMS is shown in Figure 1A. The support frame for six electromagnets is constructed from aluminum alloy, while the base is made of phenolic plastic. The electromagnets and frame are rigidly connected using aluminum alloy angle brackets and steel bolts. Figure 1B presents a cross-section of a single electromagnet, revealing its internal iron core and external composite structure: DT4E pure iron core, aluminum panel, DT4E pure iron sleeve, and DT4E pure iron cover plate. The black dashed arrow indicates the closed magnetic flux path. Furthermore, key dimensions such as core length (*L_core_*), coil length (*L_coil_*), diameter parameters (*d_max_*, *d_coil_*, *d_core_*), and core apex angle (*φ_apex_*) are shown in Figure 1C and Table 1, illustrating the compact structural design. Figure 1D presents a comparison of simulated versus measured data for a pair of electromagnets operating at 3 A current. The line plot depicts the magnetic flux density distribution near the central axis origin (−20 mm to +20 mm), with position on the horizontal axis and magnetic flux density on the vertical axis. The measured data represent the average of statistical measurements from three pairs of electromagnets. The performance verification chart indicates that the simulated and measured magnetic field curves closely match within ±15 mm, exhibiting near-linear variation. The magnetic field distribution data validate the effectiveness of the simulation design. At the origin of the working space, a single pair of electromagnets generates a magnetic field gradient of approximately 2 T/m under a 3 A current. Compared to previous electromagnetic manipulation systems, the EEMS achieves significant improvements in both magnetic field gradient and working space dimensions. Under 3 A current excitation, it achieves a magnetic field gradient of 2 T/m within a spherical working space of 106 mm diameter.

#### 3.1.3. Optimization of the Electromagnet Configuration

**Figure 2:**
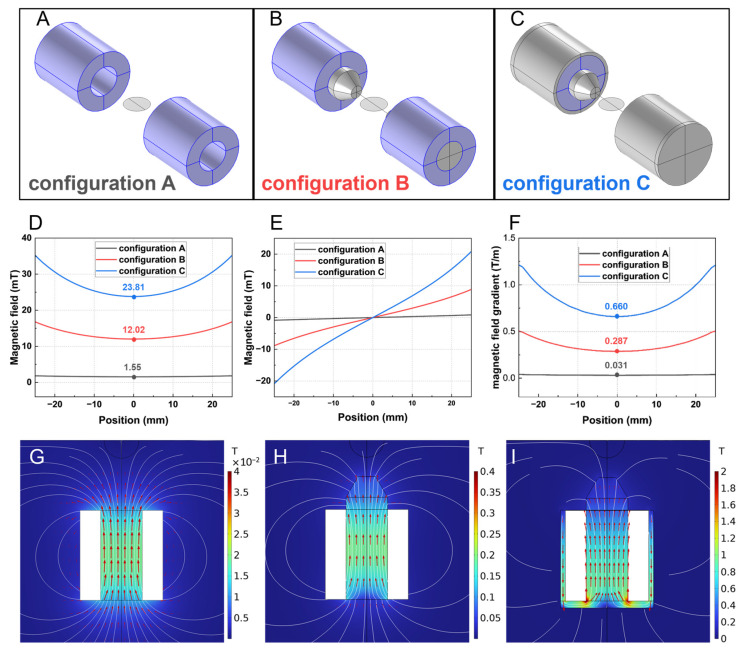
Simulation of magnetic fields from electromagnet configurations. (**A**) Configuration A: The blue area represents the coil, with no other components. (**B**) Configuration B: The blue area represents the coil, with the central gray area being the iron core. (**C**) Configuration C: The blue area represents the coil, with the central gray area being the iron core. The rear consists of a DT4E pure iron cover plate, and the exterior comprises a DT4E pure iron sleeve. (**D**) Magnetic flux density along the central axis when currents in a pair of electromagnets flow in opposite directions. (**E**) Magnetic flux density along the central axis when currents in a pair of electromagnets flow in the same direction. (**F**) Magnetic field gradient strength along the central axis when currents in a pair of electromagnets flow in the same direction. Spatial distribution of magnetic flux density in (**G**) configuration A, (**H**) configuration B, and (**I**) configuration C, the arrows indicate the direction of the magnetic field.

As shown in Figure 2A, the cylindrical coil configuration A and the iron-core-containing coil configuration B depicted in Figure 2B are common electromagnet structures for generating magnetic field gradients. Compared to configuration A, configuration B, using identical geometry, coil turns, winding specifications, and current excitation, significantly enhances both the magnetic flux density and flux density gradient within the working space. The enhanced magnetic field coil configuration C, shown in Figure 2C, employs a composite structure comprising a DT4E pure iron core, an aluminum panel, a DT4E pure iron sleeve, and a DT4E pure iron cover plate. Under 1 A current excitation in opposite directions, the magnetic flux density along the axes flanking the origin of the working space for a pair of electromagnets is shown in Figure 2D. In the simulation environment, configuration C achieves a magnetic flux density of 23.81 mT at the origin, representing 1.98 times that of configuration B and 15.36 times that of configuration A. Under a 1 A current excitation in the same direction, the magnetic flux density and magnetic flux density gradient of the magnetic field along the axes on both sides of the origin in the working space for a pair of electromagnets are shown in Figure 2E,F. In the simulation environment, the magnetic flux density gradient at the origin for configuration C is 0.660 T/m, which is 2.30 times that of configuration B and 21.29 times that of configuration A.

This series of diagrams systematically compares schematic diagrams, axial magnetic field and gradient curves, and magnetic field distribution maps to reveal the optimization mechanism of magnetic field performance through the evolution of magnetic circuits from basic coils to enhanced iron-core coils. Three configurations, bare coil, coil with internal iron core, and enhanced iron-core coil integrating a pure iron back cover plate and external pure iron sleeve, demonstrate a progressive optimization strategy in magnetic circuit design. Under identical current excitation, the enhanced iron-core coil configuration significantly outperforms the other two in both magnetic field strength and gradient along the central axis. For instance, it achieves a peak field strength of approximately 12.02 mT at specific locations. On the one hand, magnetic reluctance reduction provided by the high-permeability DT4E pure iron core increases the total magnetic flux according to Hopkinson’s law:

(12) $$\Phi =\frac{F}{R}$$

where Φ is the magnetic flux through the magnetic element, F is the magnetomotive force across a magnetic element, R is the magnetic reluctance of that element.

On the other hand, stray flux suppression is achieved by the external closed magnetic yoke, which redirects magnetic field lines into the working air gap and improves magnetic flux utilization, further increasing the magnetic field gradient in the workspace. This substantially reduces leakage flux, enabling greater magnetic flux to traverse the target working air gap. As shown in Figure 2G–I, field distribution contour plots visually validate this mechanism: the bare coil exhibits diffuse magnetic fields, while the core introduction concentrates the field toward the axis. The enhanced configuration further tightly confines the magnetic field within the pole gap, forming a high-intensity, high-gradient concentrated field zone. In the magnetic field optimization, the air area within the working space is configured as the magnetic circuit gap. The magnetic circuits outside the working space are constrained by a specific structure of DT4E pure iron with high magnetic permeability and low coercivity. This significantly reduces magnetic resistance and stray flux within the magnetic circuit, resulting in higher magnetic flux density, greater magnetic field gradient, and lower drive current.

This simulation study confirms that the composite magnetic circuit design integrating the DT4E iron core, DT4E iron sleeve, and DT4E iron cover into a complete magnetic loop is key to enhancing the capacity of the magnetic field flux density and the gradient of an individual electromagnet. By optimizing the magnetic path, it achieves synergistic enhancement of both the magnetic field flux density and gradient. This provides a crucial unit model and design basis for subsequent development of high-performance multi-electromagnet manipulation systems.

#### 3.1.4. Thermal Management of the Electromagnet

**Figure 3:**
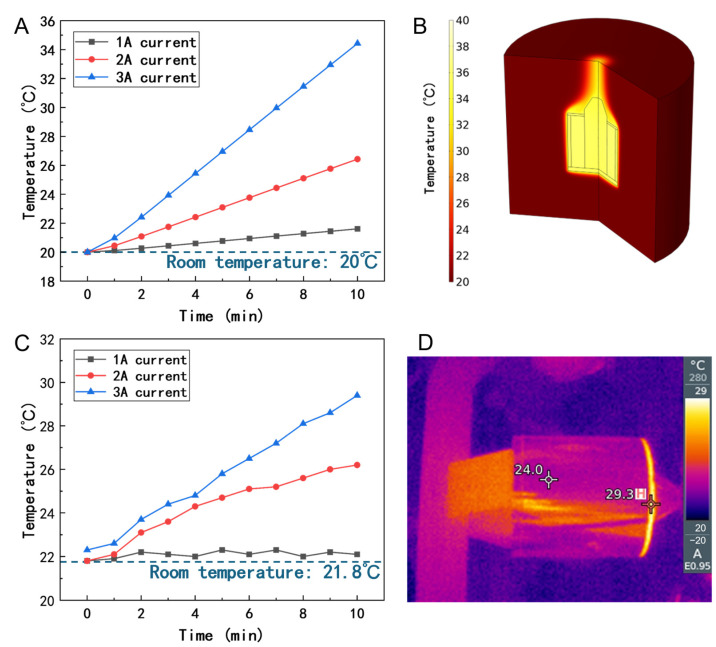
Temperature distribution of an electromagnet. (**A**) Time-dependent variation curves of the simulated maximum surface temperature under 1 A, 2 A, and 3 A current excitation. (**B**) Three-dimensional distribution of simulated temperatures for the device and surrounding environment at 10 min under 3 A current excitation. (**C**) Time-dependent variation curves of the actual measured maximum surface temperature under 1 A, 2 A, and 3 A current excitation. (**D**) Temperature distribution on the device surface measured at 10 min under 3 A current excitation.

Since the electromagnetic manipulation system is designed for biomedical applications, it must adhere to environmental temperature safety principles during operation to prevent adverse effects on manipulation targets or burn risks from excessive surface temperatures. To evaluate the system’s heat dissipation capability at room temperature, a finite element simulation was performed in COMSOL Multiphysics. The dimensions of the model were set to be the same as the actual dimensions and material properties of a single electromagnet. The ambient temperature was set to 293.15 K (20 °C), with current excitations of 1 A, 2 A, and 3 A. The time step was set to 1 min, with a total simulation duration of 10 min. The 3 A current represents the peak current for a single electromagnet. Figure 3A shows the simulated maximum temperature rise on the electromagnet surface under 1 A, 2 A, and 3 A current excitations, which were 1.6 °C, 6.4 °C, and 14.4 °C, respectively. The corresponding maximum surface temperatures were 21.6 °C, 26.4 °C, and 34.4 °C. Figure 3B presents the three-dimensional distribution of simulated device and ambient temperatures at 10 min under 3 A current excitation. The internal maximum temperature is 35.6 °C, below the normal human body temperature of 36 °C. The cylindrical bright area above the top of the electromagnet core in the figure represents convective heat dissipation airflow, with a maximum velocity of 0.29 m/s. During passive cooling, the electromagnet primarily relies on thermal conduction and convection for heat dissipation. Figure 3C shows the actual maximum surface temperature rise measured by an infrared thermometer under 1 A, 2 A, and 3 A current excitation: 0.3 °C, 4.4 °C, and 7.1 °C, respectively. The corresponding maximum surface temperatures were 22.1 °C, 26.2 °C, and 29.4 °C, all significantly below the normal human body temperature of 36 °C. We also measured the electromagnet’s surface temperature at a current of 3 A for 30 min; the temperature plot is shown in Figure S1. The initial temperature was 20.1 °C. After 30 min, the maximum surface temperature was 41.9 °C, representing a temperature rise of 21.8 °C. This indicates that the EEMS remains within safe temperature thresholds even after 30 min of continuous operation at a 3 A peak current. Its temperature variation over time follows the following fitting formula:

(13) $$T={(T}_{0}-1.2)+0.75t\;[{}^{\circ}C]\;(R^{2}>0.995)$$

where T is predicted temperature, $T_{0}$ is initial temperature, and t is operating time at peak current. Actual measured temperatures under all three current conditions were significantly lower than simulated values. This discrepancy arises because, in practice, the electromagnet is rigidly connected to the aluminum alloy bracket via aluminum alloy angle brackets and steel bolts. The inclined surface of the wedge-shaped bracket forms an interference fit with the DT4E pure iron cover plate on the back of the electromagnet. The internal Joule heating generated by the current can flow into the aluminum alloy bracket and support via thermal conduction. The aluminum alloy brackets used contain 99% aluminum, exhibiting thermal conductivity close to pure aluminum and possessing a favorable thermal conductivity coefficient. Consequently, the combined heat flux from thermal conduction and convection in actual operation exceeds simulation models. With comparable current-induced Joule heating power, the electromagnet accumulates less heat physically, resulting in a lower surface temperature rise. The selection of DT4E pure iron and aluminum for structural components provides excellent thermal conductivity. At an ambient temperature of 20 °C, DT4E pure iron has a thermal conductivity of 76.2 W/(m·K), while aluminum has a thermal conductivity of 238 W/(m·K). For comparison, the thermal conductivity of ABS plastic, a commonly used polymer structural material, is 0.209 W/(m·K), while that of PMMA plastic is 0.192 W/(m·K).

The simulation model based on Joule heating and heat transfer validated the relationship between current and temperature changes, assessed expected temperature rise under different excitation currents, and established a benchmark for selecting current parameters within the safe operation area (SOA). Subsequent comparisons between simulation data and actual measurement data revealed discrepancies between the device’s real-world thermal behavior and the simulation model, while also confirming the thermal simulation model’s potential as an effective design tool. This model can be utilized for thermal design optimization across a broader range of current parameters in subsequent stages.

### 3.2. Magnetic Manipulation System

#### 3.2.1. Magnetic Field Generation of the Electromagnet

**Figure 4:**
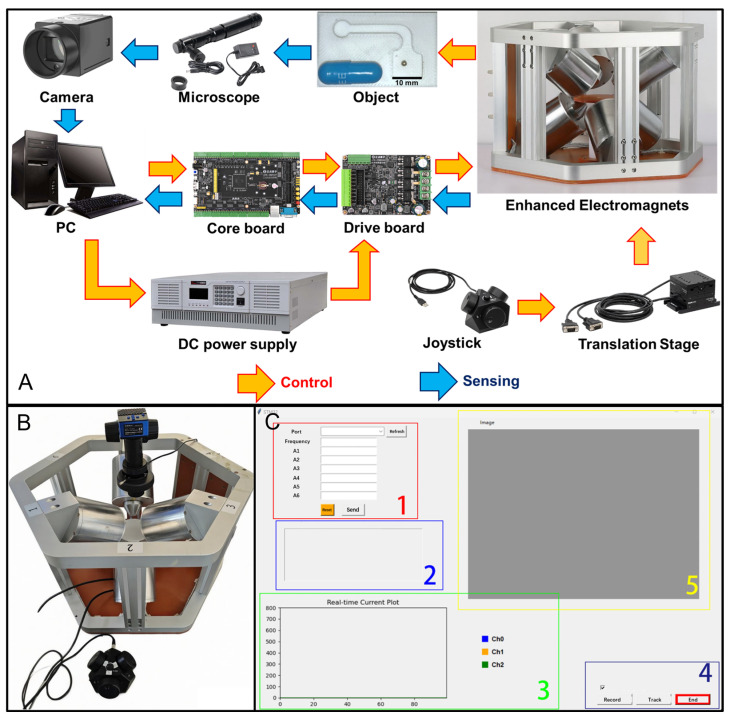
The hardware and software of the EEMS. (**A**) The EEMS hardware layer and signal chain. (**B**) The EEMS after setup completion. (**C**) The PC GUI interface: 1. Command input bar; 2. Command reception bar; 3. Real-time current graph; 4. Target tracking or video recording; and 5. Camera image frame.

The hardware layer of EEMS is illustrated in Figure 4A. The blue arrow represents the control signal chain and its direction, while the orange arrow denotes the sensing signal chain and its direction. Under working conditions, the PC, acting as the host computer, transmits control parameters to the slave device, an MCU core board based on the STM32F407 processor. The core board processes control parameters, converting them into PWM control signals sent to the driver board. The driver board’s input connects to a constant-voltage power supply, whose voltage value is controlled by the host PC. Its output consists of a PWM-signal-controlled H-bridge driver circuit. The driver board employs optocoupler isolation circuits to separate the electromagnet driver circuit from the PWM signal circuit. The driver board acquires electromagnet current data and sends it to the core board, which processes the current information before transmitting it to the host PC. The specimen stage is mounted on a 2D motorized displacement platform, controlled via a knob encoder handle. A CMOS camera is connected to the microscope, transmitting images of the objective to the computer. The host computer communicates with the slave computer and camera via USB protocol. The host computer communicates with the power supply via an RS485 protocol. The slave computer communicates with the driver board via the serial port.

Figure 4B shows the fully assembled EEMS and workspace. The host computer control panel GUI is written in Python 3.12, as depicted in Figure 4C. Box 1 displays control parameter inputs, including the communication port, rotation frequency (in constant current mode, the frequency is set to zero), peak current amplitude, and waveform phase. Box 2 shows received hardware status information. Box 3 displays acquired current data. Box 4 contains real-time target tracking and video recording buttons. Box 5 shows camera image acquisition.

#### 3.2.2. Motion Control with Magnetic Manipulation System

**Figure 5:**
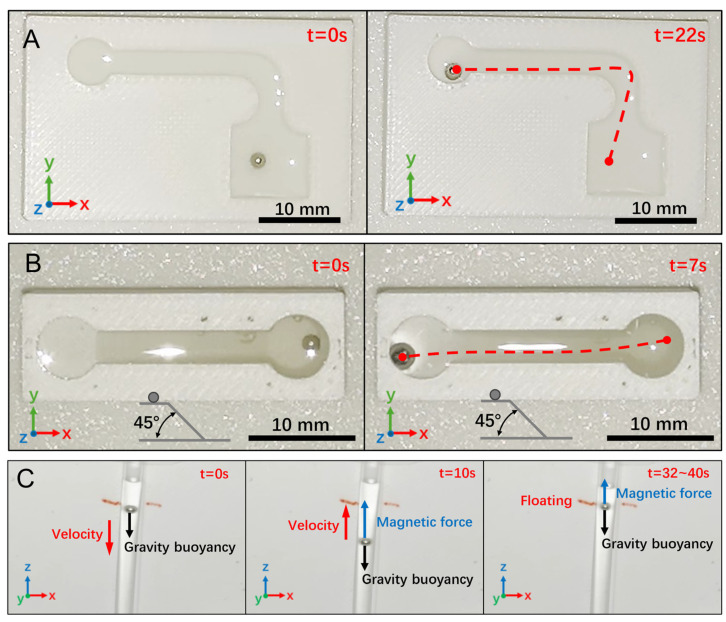
Control of microbeads using magnetic field gradient. (**A**) Magnetic beads traveling straight and turning. (**B**) Magnetic beads climbing a slope with a 45° incline. (**C**) Magnetic beads overcoming gravity to move vertically in a tube.

To validate the system’s magnetic manipulation capabilities, motion experiments were conducted using magnetic beads and nanorobotic microswarms. The beads had a diameter of 2 mm and were made of neodymium (NdFeB) magnet, with a density of approximately 7.5 g/cm^3^, close to that of iron. The applied current upper limit was capped at one-third of the maximum value, set to 0–1 A, to demonstrate the magnetic field’s performance redundancy for manipulating strong magnetic objects. Three experimental setups were configured: a horizontal displacement channel, an inclined plane climbing channel, and a vertical climbing channel. The horizontal displacement and inclined plane climbing channels were fabricated from PLA material, with the internal environment utilizing a high-viscosity 1.5% *m*/*v* sodium alginate (SA) aqueous solution. Figure 5A demonstrate straight-line travel and turning maneuvers of magnetic objects within a horizontal plane, proving the EEMS can apply precise magnetic forces and steering torques to manipulate magnetic objects, as shown in Video S1. For the 100% slope climbing experiment, the gravitational component opposing motion is mg×sin45°=0.707 mg; the magnetic force is strong enough to overcome gravity, friction, and fluid resistance. Figure 5B demonstrate magnetic object ascent along a 100% slope, indicating that the system’s magnetic driving force sufficiently overcomes gravitational and frictional forces while maintaining sustained traction capability, as shown in Video S2. The vertical ascent experiment in Figure 5C sequentially demonstrates three processes: descent without a magnetic field, magnetic force-assisted ascent, and magnetic force-balanced suspension, as shown in Video S3. During magnetic force-assisted ascent and float, the magnetic bead’s motion results from the dynamic equilibrium of multiple forces, including magnetic force, gravity, buoyancy, and fluid resistance. Magnetic field manipulation primarily overcomes gravity. We tested the ratio of magnetic force to gravitational force near the working center. When a 1 A current is applied, the magnetic force exerted on the magnet can reach 1.7 times its gravity, as shown in Figure S2. Under static levitation conditions, the drag force approaches zero and the equilibrium condition becomes $F_{mag}+F_{buo}\approx \mathrm{mg}$. At 1 A excitation current, since the maximum magnetic force exceeds the bead weight by approximately 170%, the EEMS possesses sufficient force capability for both gravity compensation and upward actuation.

**Figure 6:**
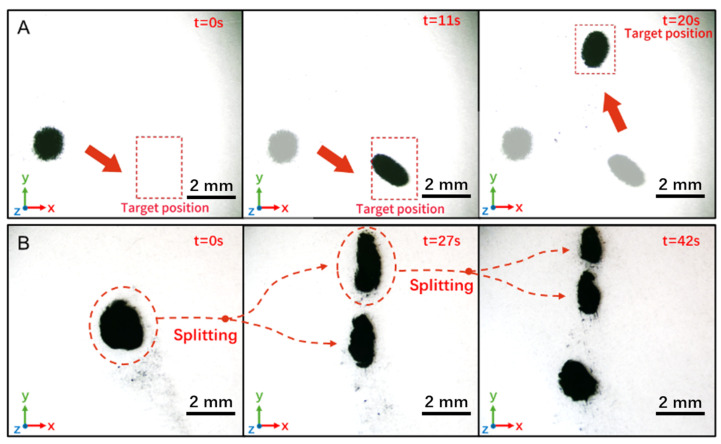
Swarm control using magnetic fields generated by the EEMS. (**A**) Magnetic microswarms moving straight and turning under the RMF. (**B**) Magnetic microswarm splitting and moving under the RMF. The arrow indicates the direction of movement.

This system can also be used to control nanorobots and microswarms. The magnetic swarm consists of Fe_3_O_4_ magnetic nanoparticles (MNPs) with a diameter of 100 nm, and the fluid environment for the experiment utilized a 10% *m*/*v* polyvinyl pyrrolidone (PVP) aqueous solution. Figure 6A demonstrate EEMS controlling the magnetic swarm for directional motion, as shown in Video S4. Firstly, EEMS creates a rotating magnetic field (RMF) within the XY plane to form a nearly disc-shaped magnetic swarm of magnetic nanoparticles. Then, a gradient magnetic field pointing toward the target region is superimposed onto the RMF, thereby driving the magnetic nanoparticles to move in a directed area. The frequency of the rotating magnetic field was 10 Hz, and the current amplitude was 1 A. The current amplitude of the electromagnet used for the gradient field is 0.5 A. The average speed of the swarm as it moved toward the target area was 0.42 mm/s. During this movement, the average area of the swarm was 1.388 mm^2^, with a maximum of 1.425 mm^2^, a minimum of 1.316 mm^2^, and a standard deviation of 0.0348 mm^2^. Figure 6B demonstrate the behavior of EEMS in controlling the magnetic swarm splitting, as shown in Video S5. EEMS generates an RMF within the XY plane, causing MNPs to form a nearly circular magnetic swarm. Subsequently, by superimposing an oscillating field of the same frequency as the RMF in the target direction, this strategy deforms the magnetic swarm into an elliptical disc with its major axis aligned along the oscillating field direction, splitting it into two smaller magnetic swarms. Before division, the swarm had an area of 2.739 mm^2^. After the first division, it split into two swarms with areas of 2.052 mm^2^ and 1.745 mm^2^, bringing the total area of the swarms to 3.797 mm^2^. After the second division, three swarms with areas of 1.165 mm^2^, 1.298 mm^2^, and 1.877 mm^2^ were formed, and the total area of the swarms increased to 4.339 mm^2^, representing a 58% increase compared to the initial area. These experiments demonstrate the versatility of the EEMS to achieve complex control of microswarms through the simultaneous use of RMF, oscillating field, and gradient field.

**Figure 7:**
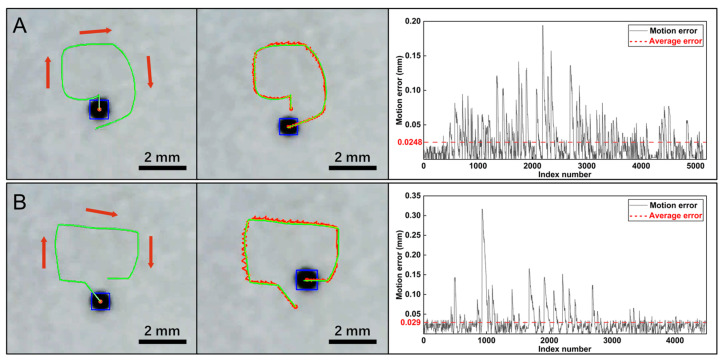
Closed-loop motion control using magnetic fields generated by the EEMS. (**A**) Magnetic microspheres moved with a nearly circular path. (**B**) Magnetic microspheres moved with a nearly rectangular path. The arrow indicates the direction of movement

In order to evaluate the closed-loop motion control performance of the EEMS for magnetic targets, motion experiments were conducted using sodium alginate (SA) magnetic microspheres as the manipulated objects. The SA magnetic microspheres have a diameter of approximately 900 micrometers and a density of approximately 1.2 g/cm^3^. In order to demonstrate magnetic drive capability in a viscous fluid, the fluid environment in the microchannel constituted a 1% *m*/*v* guar gum solution. The magnetic microspheres were then transferred onto a glass slide that had been coated with a circular PDMS flow channel, using a pipette. A vision-feedback-based PID controller was employed to perform closed-loop control of the microspheres’ automatic motion, the schematic diagram of the control algorithm is shown in Figure S5. The green planned path was generated by means of a mouse pointer on the XY plane, which was tracked by a vision sensor on the host computer. The program successfully tracked the geometric center of the microsphere, automatically controlled its motion from the initial position to the final position of the planned path, and calculated the positional error between the red actual path and the green planned path. For the planned path, which is nearly circular (Figure 7A), the average motion error was 0.0248 mm, the standard deviation was 0.0284 mm, and the maximum motion error was approximately 0.19 mm. For the rectangular path in Figure 7B, the mean error is 0.029 mm, the standard deviation is 0.0368 mm, and the maximum motion error is approximately 0.32 mm. In the closed-loop motion experiments, the average motion error of the sodium alginate microspheres was less than 0.03 mm. This indicates that the EEMS can achieve sub-millimetre motion accuracy when manipulating millimetre-scale targets.

Collectively, these experiments demonstrate the system’s core capabilities: high-precision spatial guidance for micro/nanomanipulation and robust load-carrying capacity. This endows it with clear application potential in cutting-edge fields requiring complex three-dimensional non-contact manipulation, such as targeted therapy and robotics at the milli-, micro-, and nanoscale.

## 4. Discussion

In this work, an enhanced electromagnetic manipulation system (EEMS) was designed, implemented, and systematically evaluated to address the long-standing trade-off between magnetic field strength, field gradient, and usable workspace in conventional electromagnetic actuation platforms. The central innovation of this study lies in prioritizing magnetic circuit optimization through the integration of high-permeability structural materials and refined geometric design, rather than relying solely on coil arrangement or control strategies. The resulting system, composed of six identically structured electromagnets arranged in a rotated Cartesian configuration, enables the generation of both uniform and gradient magnetic fields within a spherical workspace of 106 mm in diameter. Finite element simulations and experimental measurements demonstrate strong agreement, confirming the reliability of the proposed design methodology. The EEMS achieves magnetic flux densities up to 300 mT and magnetic field gradients up to 9.5 T/m within the workspace, representing a substantial performance improvement compared to existing systems with similar spatial dimensions. Furthermore, thermal analyses and infrared measurements verify that the system can operate continuously without active cooling, owing to efficient magnetic flux guidance and enhanced heat dissipation provided by DT4E pure iron and aluminum alloy components. Magnetic bead motion experiments and magnetic swarm control experiments conducted under various orientations further validate the system’s flexible, precise controllability, and adaptability in complex mission environments.

The proposed EEMS provides a high-performance and versatile magnetic field generation platform that bridges the gap between strong magnetic actuation, long operational time through efficient thermal management, and enhanced large working space accessibility. This system establishes a solid foundation for future research in micro/nanorobotics, targeted biomedical manipulation, and intelligent magnetic systems. Future work will focus on quantitative dynamic modeling of microrobot motion, closed-loop control integration, and the extension of the platform toward multi-agent and three-dimensional autonomous magnetic manipulation.

## Figures and Tables

**Table 1:** Parameters of a single electromagnet.

Symbol	Definition	Value
*n*	Wraps	2380
*d_w_*	Wire diameter	1 mm
*L_coil_*	Coil length	130 mm
*L_core_*	Core length	180 mm
*d_coil_*	Coil diameter	120 mm
*d_core_*	Core diameter	60 mm
*d_max_*	Max diameter	140 mm
*φ_apex_*	Optimized apex angle	60°
*d_wp_*	Workplace diameter	100 mm
*r*	Coil resistance	14 Ω

## Data Availability

The dataset is available upon request from the authors.

## Supplementary Materials

The following supporting information can be downloaded at https://www.mdpi.com/article/10.3390/mi17070810/s1, Table S1. Parameters of the thermal simulation for an electromagnet. Figure S1. The plot of the maximum surface temperature over 30 min. Figure S2. (A) The weight of the magnet is 0.40 g when the EEMS magnetic field is not activated. (B) When applied to a downward magnetic field generated by a 1 A current, the force on the magnet is equivalent to the mass of a 1.11 g object. Figure S3. Cross sectional Distribution of the Magnetic Field in the Working Space When a 1A Current Flows Through a Single Electromagnet. Figure S4. Magnetic field gradient along the central axis of three representative extreme geometric shapes. (A) 60° beveled tip; (B) conical tip; (C) cylindrical tip. Figure S5. Schematic diagram of the control solving algorithm. Video S1: Straight-line travel and turning maneuvers of the magnetic object. Video S2: Experiment of the magnetic object ascent along a 100% slope. Video S3: Experiment of vertical ascent and floating for the magnetic object. Video S4: Directional motion experiment for the magnetic swarm. Video S5: Magnetically controlled splitting experiment for the magnetic swarm.

## Abbreviations

The following abbreviations are used in this manuscript:

EEMS	Enhanced electromagnetic manipulation system
MNPs	Magnetic nanoparticles
SOA	Safe operation area
SA	Sodium alginate
PVP	Polyvinyl pyrrolidone
RMF	Rotating magnetic field
