# Supplementary material for: An Enhanced Electromagnetic Manipulation System with a Large Workspace, High-Gradient Magnetic Actuation, and Efficient Thermal Management
Source: Micromachines (Basel). 2026 Jul 2;17(7):810. doi: 10.3390/mi17070810 (PMC13414223; doi:10.3390/mi17070810)
Supplement: Supplementary file 1 [file micromachines-17-00810-s001.zip › micromachines-4363551-supplementary/Supplementary Materials.pdf]

Supplementary Materials

Table S1. Parameters of the thermal simulation for an electromagnet.

Symbol	Quantity	Value
V_{coil}	Coil volume	0.97 dm^3
η_f	Wire filling rate	0.7
V_{wire}	wire volume	0.679 dm^3
η_c	Wire copper rate	0.76
V_{copper}	copper volume	0.513 dm^3
T_r	Heat resistant	403.15 K
P_{1A}	Power at 1 A current	14 W
P_{2A}	Power at 2 A current	56 W
P_{3A}	Power at 3 A current	126 W
r	Coil resistance	14 Ω
V_{Ins}	Insulation voltage	500 V
T_{lim}	Temperature limit	130 °C
I_{lim}	Current limit	3 A

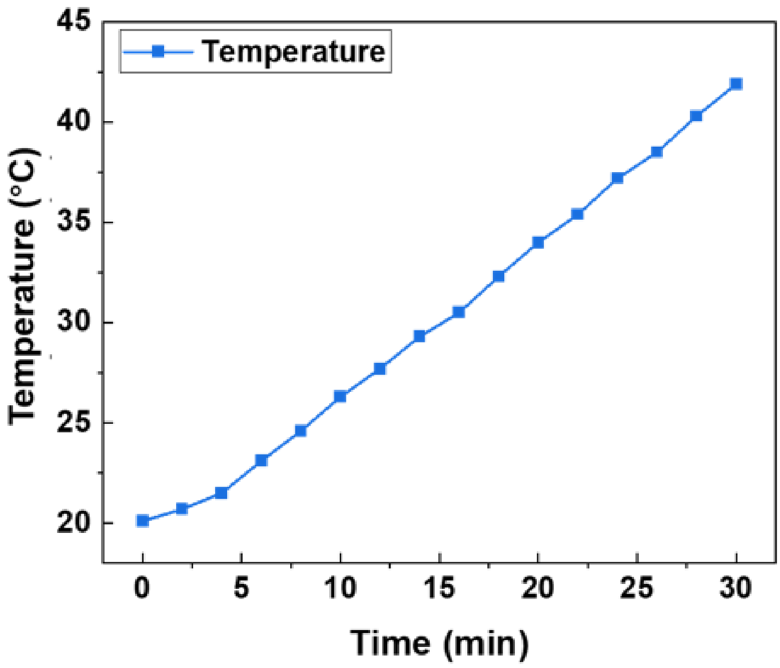

Figure S1. The plot of the maximum surface temperature during 30 min.

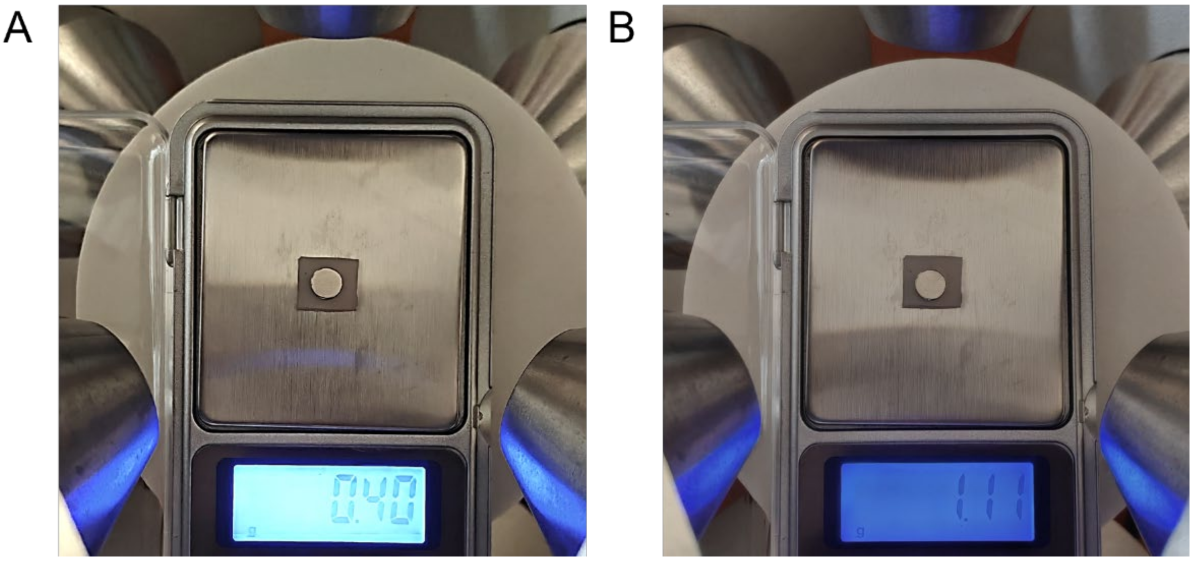

Figure S2. (**A**) The weight of the magnet is 0.40g when the EEMS magnetic field is not activated. (**B**) When applied to a downward magnetic field generated by a 1A current, the force on the magnet is equivalent to the mass of a 1.11g object.

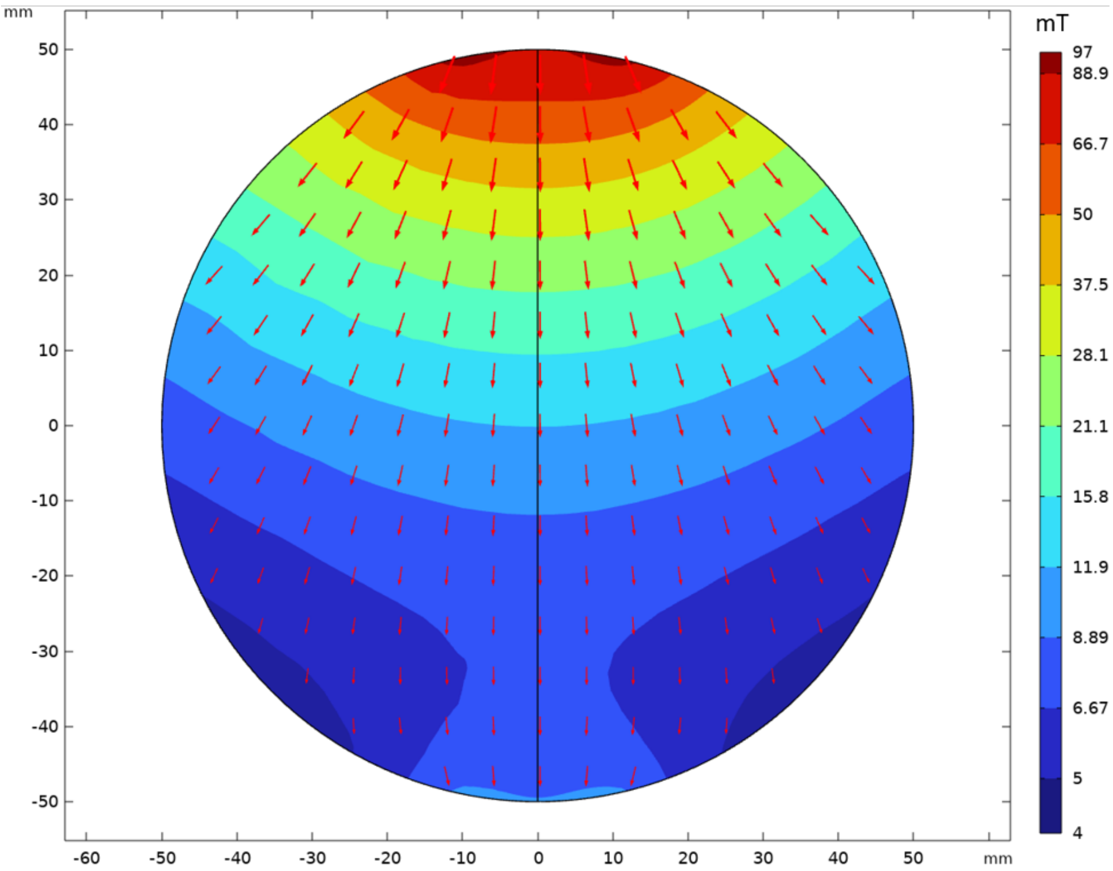

Figure S3. Cross-sectional Distribution of the Magnetic Field in the Working Space When a 1A Current Flows Through a Single Electromagnet.

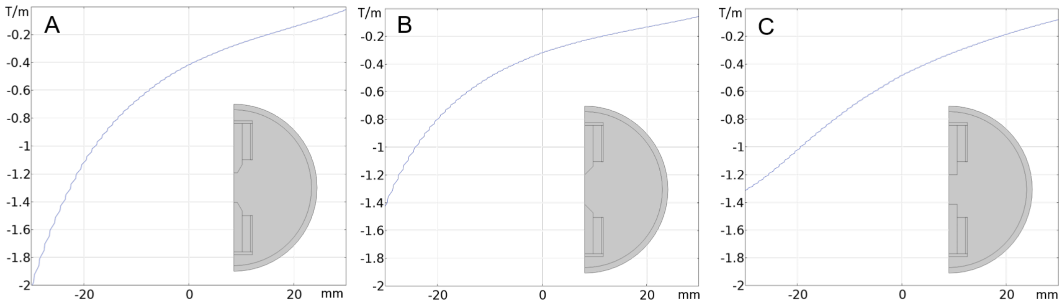

Figure S4. Magnetic field gradient along the central axis of three representative extreme geometric shapes. (**A**) 60° beveled tip; (**B**) conical tip; (**C**) cylindrical tip.

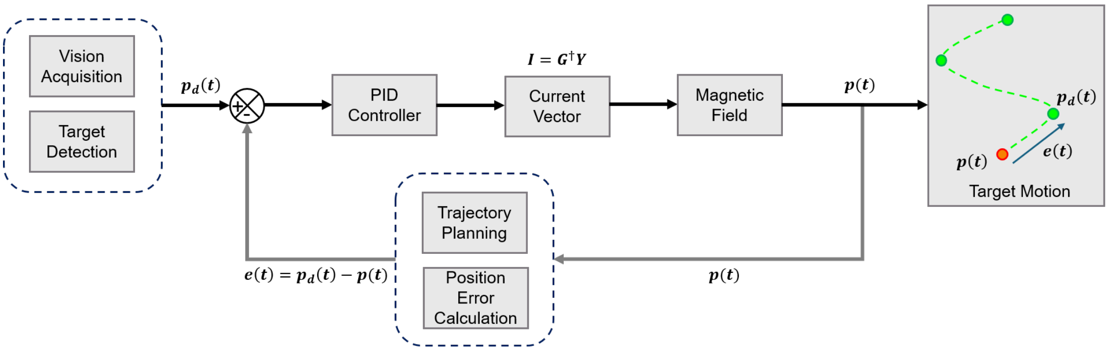

Figure S5. Schematic diagram of the control solving algorithm.

Under low-Reynolds-number conditions, the Stokes drag act on a spherical particle scale linearly with particle radius,

$$F_{drag} = 6\pi\eta r v \tag{1}$$

where F_{drag} is fluid drag (N), η is dynamic viscosity of the fluid (Pa·s), r is radius of the microsphere (m), v is velocity of the microsphere relative to the fluid (m/s).

When the velocity v and viscosity η are constant,

$$F_{drag} \propto r \tag{2}$$

For microsphere that are not fully magnetized, magnetic force experienced in a magnetic field is as follows:

$$F_{mag} = V\chi \frac{B\nabla B}{\mu_0} \tag{3}$$

where F_{mag} is magnetic force (N), $V = \frac{4}{3}\pi r^3$ is volume (m^3), χ is magnetic susceptibility, B is magnetic field (T), ∇B is magnetic field gradient (T/m), μ_0 is vacuum permeability.

Substitute the volume formula into the above equation,

$$F_{mag} \propto r^3\chi \tag{4}$$

When considering density,

$$G \propto r^3(\rho - \rho_0) \tag{5}$$

where G is gravity (N), ρ is density of microspheres (kg/m^3), ρ_0 is density of fluid (kg/m^3).

The ratio between magnetic force and fluid drag is as follows:

$$\frac{F_{mag}}{F_{drag}} \propto r^2\chi \tag{6}$$

which indicates that the magnetic actuation capability increases proportionally to $r^2\chi$, implying that larger particles or particles with higher magnetic loading are generally easier to manipulate under identical field conditions.

The ratio between magnetic force and gravity is as follows:

$$\frac{F_{mag}}{G} \propto \frac{\chi}{(\rho - \rho_0)} \tag{7}$$

which is independent of particle size. Therefore, particles possessing similar magnetic susceptibility and density exhibit comparable gravitational compensation capability regardless of their absolute dimensions.
